# The implementation of community-based programs in Vietnam is promising in promoting health

**DOI:** 10.3389/fpubh.2023.1182947

**Published:** 2023-06-20

**Authors:** Zinzi E. Pardoel, Sijmen A. Reijneveld, Robert Lensink, Maarten Postma, Tran B. Thuy, Nga C. Viet, Lien N. T. Phuong, Jaap A. R. Koot, Jeanet J. A. Landsman

**Affiliations:** ^1^Department of Health Sciences, University Medical Center Groningen, University of Groningen, Groningen, Netherlands; ^2^Faculty of Economics and Business, University of Groningen, Groningen, Netherlands; ^3^Department of Pharmacology and Therapy, Faculty of Medicine, Universitas Airlangga, Surabaya, Indonesia; ^4^Centre of Excellence in Higher Education for Pharmaceutical Care Innovation, Universitas Padjadjaran, Bandung, Indonesia; ^5^HelpAge International, Hanoi, Vietnam

**Keywords:** community-based programs, community-based health promotion, ageing, health promoting activities, positive health, older people associations, RE-AIM evaluation framework

## Abstract

**Background:**

Low-and middle-income countries mostly have ageing populations with many unmet economic, social, or health-related needs, Vietnam being an example. Community-based support in Vietnam, organized as Intergenerational Self-Help Clubs (ISHCs) based on the Older People Associations (OPA) model, can help to meet these needs by the provision of services for various aspects of life. This study aims to assess the implementation of the ISHCs and whether successful implementation is associated with more member-reported positive health.

**Methods:**

We used the RE-AIM (*Reach, Effectiveness, Adoption, Implementation, and Maintenance*) framework to evaluate the implementation using multiple data sources: ISHC board surveys (*n* = 97), ISHC member surveys (*n* = 5,080 in 2019 and *n* = 5,555 in 2020), focus group discussions (6; *n* = 44), and interviews with members and board leaders (*n* = 4).

**Results:**

*Reach* ranged between 46 and 83% of ISHCs reaching target groups, with a majority of women and older people participating. Regarding *Effectiveness*, members indicated high satisfaction with the ISHCs. *Adoption* scores were high, with 74%–99% for healthcare and community support activities, and in 2019, higher adoption scores were associated with more members reporting good positive health. In 2020, reported positive health slightly decreased, probably due to the influence of the COVID-19 pandemic. A total of 61 ISHCs had consistent or improving *Implementation* from 2019 to 2020, and confidence in *Maintenance* was high.

**Conclusion:**

The implementation of the OPA model in Vietnam is promising regarding its promotion of health and may help to tackle the needs of an ageing population. This study further shows that the RE-AIM framework helps to assess community health promotion approaches.

## 1. Introduction

Community-based support for older people in low-and middle-income countries has proven to be promising in the promotion of wellbeing and socio-economic circumstances of older people (1). Ageing is a major global trend that transforms societies and healthcare around the world. This demographic trend proceeds more rapidly in low-income countries than in high-income countries (2) due to declining fertility and mortality as well as increased life expectancy (3). In the context of low-and middle-income countries, resources are often scarce, and policies and care to be provided are not yet specified for older populations, resulting in several challenges such as poor access to and availability of healthcare for older people (4). Vietnam is one of the top 10 countries that experiences the highest rate of population ageing (5, 6), with 12% of the total population being aged 60 years and older, and by 2050 this is expected to rise to more than 25% (7). The majority of older people have unmet economic, social, or health-related needs (8).

**Figure 1:**
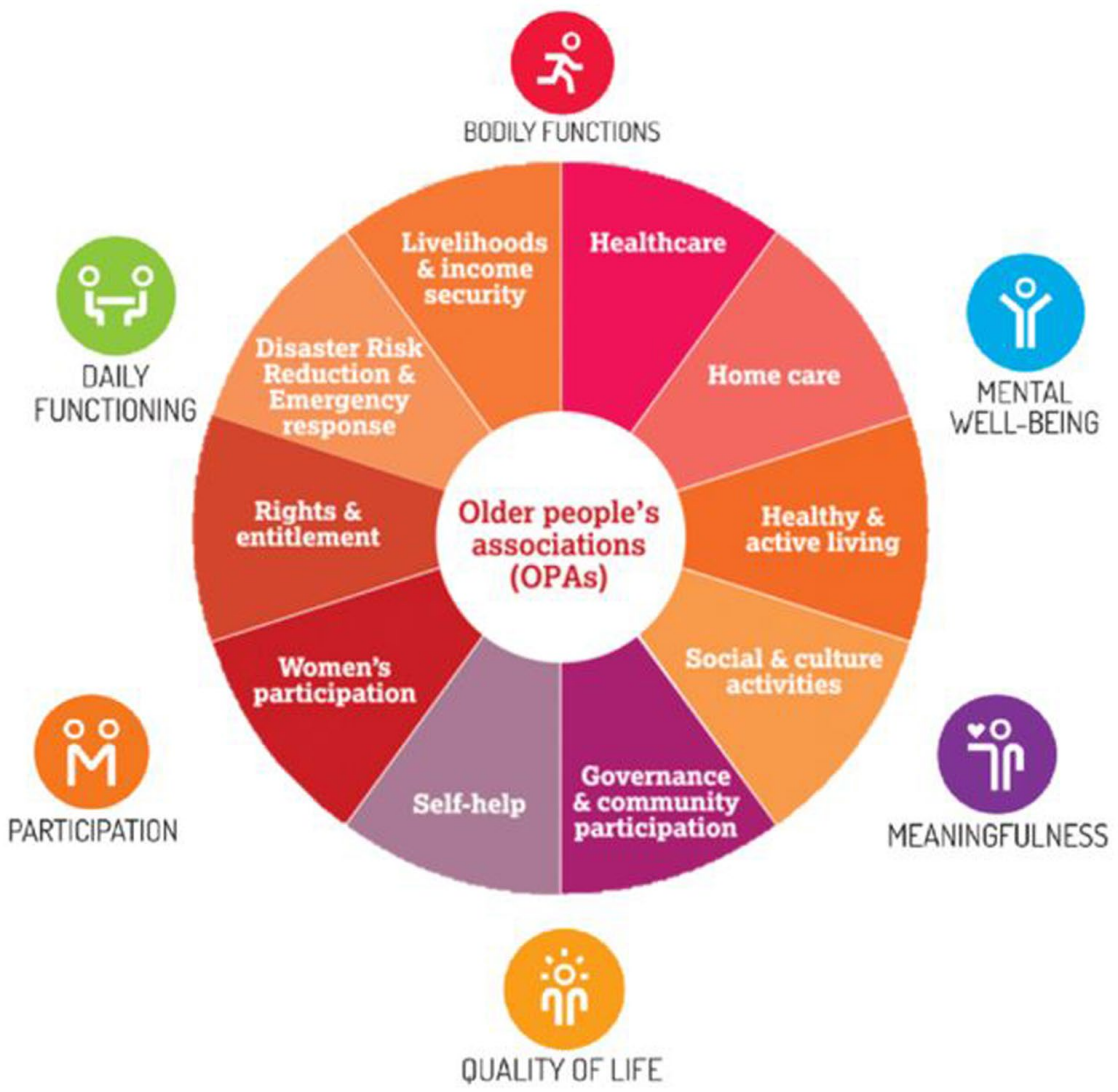
Overview of activities and services provided by OPAs within the concept of positive health (10, 11).

To meet the needs of an increasing group of older people, the concept of Older People Associations (OPAs) has been implemented throughout Southeast Asia (9). OPAs have a holistic approach, utilizing the resources and skills that older people have to provide effective social support and facilitate activities. OPAs focus on 10 overarching core areas, as shown in Figure 1; the OPAs’ implementation is country specific. The theoretical basis of the OPAs relates to the concept of “Positive Health” (see Figure 1) (11). According to this positive health model, bodily functioning, mental wellbeing, meaningfulness, quality of life, participation, and daily functioning are all dimensions that establish personal health. The multifunctional approach of the OPAs is to organize activities and provide services to meet all of these “Positive Health” dimensions. After the initial success of OPAs in Cambodia in enhancing living conditions (8), neighboring countries started applying this model as well (12).

In Vietnam, OPAs are implemented as Intergenerational Self-Help Clubs (ISHCs) (13), which are co-created with the target groups, focusing on the potential of the areas and specific challenges of the community following the dimensions of positive health (12). The ISHC is a national model covering all provinces. Community members become engaged via community-orientated activities, i.e., village meetings or loudspeaker announcements. Five members are chosen per ISHC by other members to form the management board, and priority is given to female and older people and to people with economic or social disadvantages. The ISHCs receive support and resources from NGOs and local funds, such as training and equipment, to carry out activities, and they collaborate with commune health stations regarding health activities, such as screening. Their area of focus is determined in consultation with the members. ISHCs promote multiple aspects, such as psychosocial health, healthy and active lifestyles, economic development, rights and entitlements, and self-help and peer support, i.e., helping each other in the community and improving members’ livelihoods. Moreover, ISHCs organize social, cultural, and self-reliance activities and offer legal support and homecare volunteer-based services. The ISHCs can have effects on health if implemented properly. Relevant aspects of the implementation of ISHCs include reaching community members and specific target groups, representativeness, the perceived effects for the members, embracement of the ISHCs in the communities, implementation consistency, becoming part of routine organizational practices, and maintaining effectiveness. Apart from gray literature evaluations (14), scientific evidence on how ISHCs achieve this is mostly lacking.

**Figure 2:**
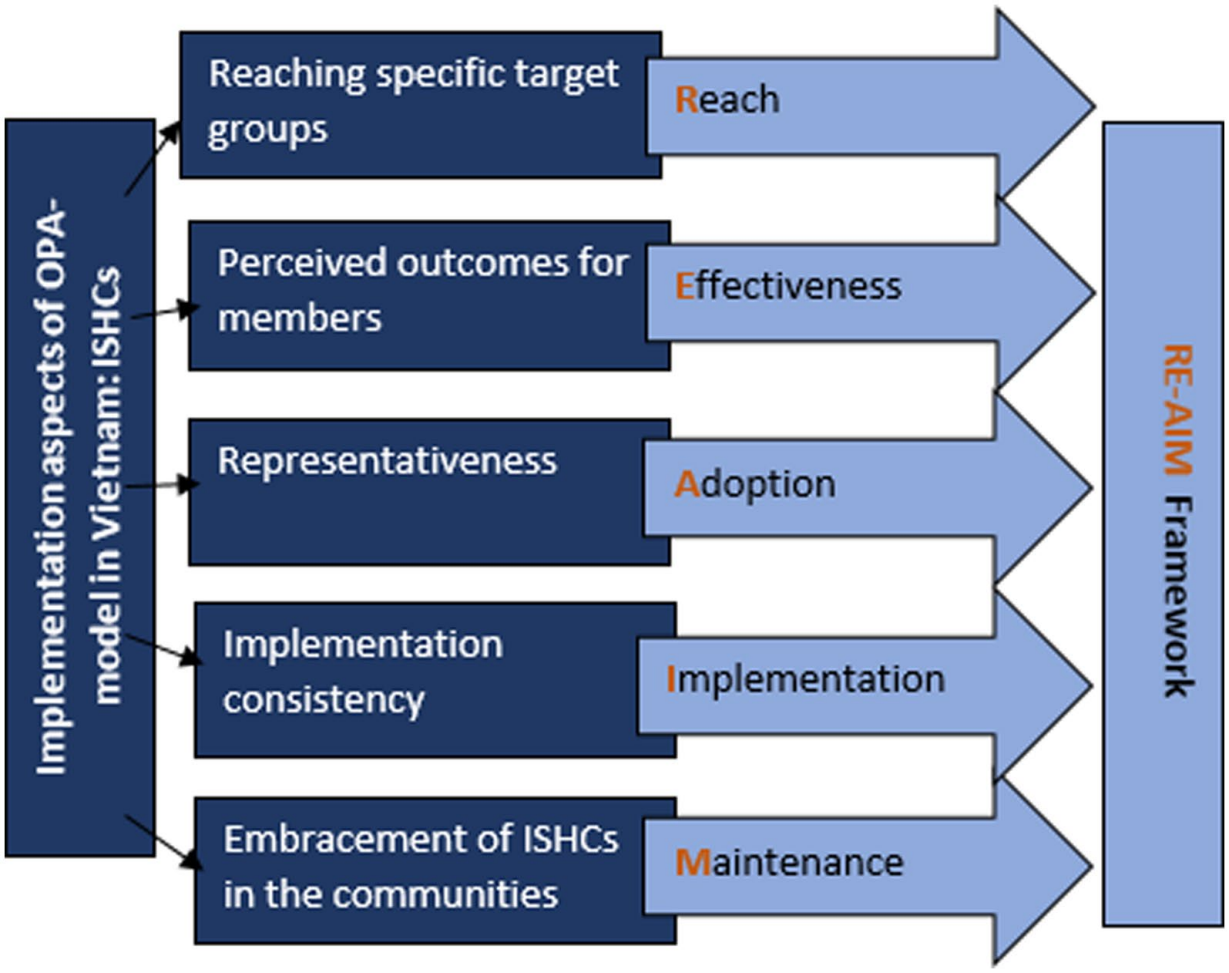
The relationship between implementation aspects of ISHCs and the RE-AIM framework.

The ISHCs’ implementation aspects align with the dimensions of the RE-AIM framework (15). The RE-AIM framework is a planning and evaluation framework that addresses dimensions of individual- and organizational-level outcomes that relate to program impact and sustainability, namely: Reach, Effectiveness, Adoption, Implementation, and organizational and individual Maintenance (16) (see Figure 2). The RE-AIM framework has been developed to address issues, dimensions, and steps in the implementation process that can facilitate or impeded success and translate scientific evidence for public health and policy (15). RE-AIM includes a focus on the design, dissemination, and implementation process that can either facilitate or impede success in achieving a broad and equitable population-based impact, which aligns with the OPA-model. This study has two aims: to assess (1) the implementation of the ISHCs with the use of the RE-AIM framework and (2) whether successful implementation is associated with more members reporting good positive health.

## 2. Methods

### 2.1. Study design

This mixed-method study was conducted among 97 ISHCs in nine provinces in the northern region of Vietnam in 2019 and 2020. Around 80% of the ISHCs were located in the countryside, 11% in an urban or semi-urban area, and 9% in a mountainous area. These ISHCs were established one to three years before the study took place. ISHC management board surveys and member surveys were conducted, and focus groups discussions (FGDs) and interviews were held with members and group leaders. Data were collected by HelpAge International (HAI), an international NGO involved in the implementation of the ISHCs. Multiple data sources were assessed to study the individual- and organizational-level outcomes of the ISHCs in 2019 and 2020 with both quantitative and qualitative data, yielding in-depth evidence on the RE-AIM and positive health dimensions. We applied the RE-AIM framework to address the first aim with ISHCs board surveys, FGDs, and interviews and the concept of positive health to address the second aim with the members surveys.

### 2.2. Sample and procedure

We assessed the two aims using four data sources, namely, ISHC management board surveys, member surveys, focus groups discussions (FGDs), and interviews.

#### 2.2.1. ISHCs management and board surveys

The first source, the ISHCs management board surveys, incorporated surveys from 97 ISHCs performed in 2019 and 2020. These surveys addressed the performances of the ISHCs, i.e., whether each club reached the targets that were set by HAI and the Vietnamese Association of the Elderly (VAE), a social organization that collaborates with HAI and supports the ISHCs. The survey had 19 indicators with specific targets (see Supplementary Table S1 for targets) that were set to ensure the ISHCs could operate sustainably and independently without support from HAI and VAE.

#### 2.2.2. Members surveys

The second source, the members surveys, consisted of cross-sectional structured surveys of members of the same 97 ISHCs in 2019 (*n* = 5,080) and 2020 (*n* = 5,555). The questions addressed satisfaction with and outcomes of ISHCs membership regarding organized activities, such as *“On a scale of 0(=not at all) to 10(=very) how much do you like the monthly organized meeting?*,” and positive health, such as “*Do you think your health behavior changed due to participation in the ISHC?*” All ISHC members were invited to participate in the survey. All questions of the survey concerned the previous 12 months of membership, and therefore, participants were included if they were a member for at least 1 year. Table 1 provides an overview of the background characteristics of the members who filled in the surveys. The average age was 60 years, and in total, 22% in 2019 and 23% in 2020 were male. Two to 8 % had a disability (2019: 8% and 2020: 2%), and almost 70% were poor to near poor (2019: 66% and 2020: 68%).

**Table 1:** Background characteristics of member surveys in 2019 and 2020 per province.

Provinces^a^	1	2	3	4	5	6	7	8	9	Total
Year	2019									
Male *n* (%)	89 (18)	137 (26)	144 (26)	129 (22)	165 (25)	131 (16)	130 (22)	83 (22)	124 (24)	1,123 (22)
Age μ (range)	60.2 (27–80)	59.4 (23–82)	61.5 (26–91)	62 (25–84)	56.4 (26–82)	60.5 (21–86)	60.3 (28–89)	64.4 (26–87)	58.7 (26–87)	60.2 (21–91)
Not/slightly disabled *n* (%)	484 (98)	480 (92)	464 (83)	497 (93)	639 (97)	730 (89)	519 (88)	333 (88)	515 (98)	4,611 (92)
Poor to near poor *n* (%)	488 (99)	329 (63)	379 (68)	412 (74)	442 (67)	495 (60)	365 (62)	306 (81)	350 (67)	3,367 (66)
Year	2020									
Male *n* (%)	115 (22)	145 (27)	149 (26)	123 (20)	164 (24)	184 (21)	146 (24)	146 (23)	126 (24)	1,298 (23)
Age *μ* (range)	60.6 (31–81)	60.9 (24–83)	62.3 (14–92)	63 (24–87)	57.7 (27–83)	60.1 (28–86)	61.2 (29–90)	62.7 (27–90)	60.1 (27–81)	60.9 (14–92)
Not/slightly disabled *n* (%)	513 (99)	538 (99)	540 (94)	594 (98)	654 (97)	854 (99)	601 (99)	623 (97)	528 (99)	5,445 (98)
Poor to near poor *n* (%)	358 (69)	390 (72)	384 (67)	414 (68)	392 (58)	609 (71)	421 (69)	470 (73)	357 (67)	3,794 (68)

a 1. Bac Ninh (*n* = 495 2019/*n* = 517 2020)/ 2. Hai Duong (*n* = 521 2019/*n* = 540 2020)/ 3. Hai Phong (*n* = 558 2019/*n* = 570 2020)/ 4. Hanoi (*n* = 536 2019/*n* = 609 2020)/ 5. Hoa Binh (*n* = 658 2019/*n* = 677 2020)/ 6. Hung Yen (*n* = 822 2019/*n* = 861 2020)/ 7. Ninh Binh (*n* = 538 2019/*n* = 608 2020) /8. Thai Binh (*n* = 379 2019/*n* = 643 2020)/ 9.Vin Phuc (*n* = 524 2019/*n* = 530 2020).

#### 2.2.3. Focus group discussions and interviews

The third and fourth sources were six FDGs (*n* = 44) and four interviews (*n* = 4), respectively. The FGDs were held with a total of 44 participants, ranging between 3 and 12 participants per discussion and with ages between 53 years and 80 years. Participants were included if they were members, group leaders, or volunteers at ISHCs from a coastal area, a mountainous area, a rural area, an urban area, or two semi-urban areas. In total, there were 23 male and 21 female participants. The topics discussed in the FGDs were experiences and satisfaction of the members with participation, changes in life due to participation, barriers and facilitators to participation in the clubs, and future perspectives on their participation. The interviews were held with two chairpersons of the VAE, a health center official and an official at the Vietnamese Fatherland Front. Participants were included based on their involvement in the organization and management of the clubs. The topics discussed in the interview covered experiences and perspectives on the organization, management, and sustainability of the ISHCs. The interviews and FGDs were held in 2019.

### 2.3. Measures

Table 2 gives an overview of the RE-AIM dimensions used to address the first aim, displaying the definition, data source, and operationalization of each dimension. We measured the dimension **Reach** by describing to what extent the ISHCs reached 70% female, 70% older, and 70% poor or disadvantaged people. Moreover, we measured **Effectiveness** in the qualitative studies as satisfaction with ISHCs, including rating satisfaction with participation in the ISHCs on a scale of 0–10 (0 = not satisfied at all, 10 = very satisfied). We measured the dimension **Adoption** as the extent to which the ISHCs achieved the targets set by VAE and HAI (see Supplementary Table S1 for targets). Furthermore, we measured **Implementation** as the differences in achieved targets over 2019 and 2020. Finally, we measured **Maintenance** as the experiences within the interviews (organizational level) with chairpersons and group leaders on the topics of management and sustainability and within the focus group discussions (individual level) with members on the topics of personal experiences and willingness to remain as members.

**Table 2:** The included variables and operationalization per RE-AIM dimension and the used data source for the first aim.

Dimension	Definition	Data source	Operationalization
Reach	Types of people who participated in the programs, also defined as the extent to which the target groups were covered.	ISHCs surveys	70–70-70% formula reached per province.
Effectiveness	Members’ experiences with participating in the club.	Focus group discussions	Satisfaction and experiences with ISHCs.
Adoption	Defined as characteristics of the ISHCs and description of ISHCs reaching targets.	ISHC surveys	Description ISHCs achieving targets on 16 indicators.^a^
Implementation	The extent to which the ISHCs were consistently implemented over 2 years.	ISHC surveys	Description of differences in achieved targets for adoption between 2019 and 2020.
Maintenance	Organizational level: defined as evidence of embedding ISHCs into routine operations and budgets.	Interviews	Description of experiences with sustainability and management.
	Individual level: defined as evidence of sustaining benefits and participants’ intentions to continue the program.	Focus group discussions	Description of personal experiences and willingness to retain membership of ISHCs.

a See Supplementary Table S1 for targets set for the 16 indicators.

Table 3 gives an overview of the positive health dimensions used to address the second aim, displaying the belonging aspects and operationalization of each dimension. We measured **Bodily functioning** by perceived improved health behavior and improved health status, which were recoded into *improved health*, **Sense of purpose** by improved perceived *level of confidence*, **Participation** by perceived *improved feeling of unity/solidarity*, **Quality of life** by perceived *improved quality of life*, **Mental wellbeing** by *disability status* (in hearing, seeing, mobility, remembering, focus, self-care, and communication) and **Daily functioning** by perceived *improved rights and entitlement*. All the variables were dichotomized. We further computed a summary measure of positive health by summing the scores on all the dimension variables and then dichotomizing these using the median as the cut-off, with a score equal to the median and above indicating good health. The variable *Good Positive Health* was created by the proportion of members reporting good positive health per ISHC. The implementation aspects *Reach* and *Adoption* were created by a summary measure of the operationalized indicators. *Implementation* was measured by the change of the overall adoption score from 2019 to 2020, which was categorized into decline (=0) and consistent or improvement (=1).

**Table 3:** The included variables and operationalization per positive health dimension for the second aim.

Positive health dimension	Belonging aspects	Operationalization^a^
Bodily functioning	Feeling healthy, physical condition, sleeping pattern, exercising, eating pattern	Perceived improved health behavior and improved health status, recoded into *improved health*
Sense of purpose	Feeling confident, accepting life, wanting to achieve ideals	Perceived improved *level of confidence*
Participation	Social contacts, being taken seriously, support of others, belonging	Perceived *improved feeling of unity/solidarity*
Quality of life	Enjoyment, being happy, feeling good, well-balanced, safe	Perceived *improved quality of life*
Mental wellbeing	Being able to remember things, to concentrate, communicate, handle changes, and having control,	D*isability status* (in hearing, seeing, mobility, remembering, focus, self-care, and communication)
Daily functioning	Knowledge about own limitations, health, money, time management, being able to ask for help	Perceived *improved rights and entitlement*.

a The dimensions of positive health were measured with the members surveys.

### 2.4. Analysis and reporting

#### 2.4.1. Qualitative analysis and reporting

First, we reported on the implementation of the ISHCs following the dimensions of the RE-AIM framework. The dimensions *Effectiveness* and *Maintenance* were qualitatively analyzed by content analysis of the FGDs and interviews, in which the data were categorized, grouped, coded, and themed under effectiveness, positive and negative experiences, expectations, maintenance, and sustainability. Qualitative analyses were carried out with Atlas.ti 23.

#### 2.4.2. Quantitative analysis and reporting

The dimensions Reach, Adoption, and Implementation were quantitatively analyzed using the management board surveys and members surveys of the ISHCs. Second, we analyzed whether successful implementation was associated with more members reporting good positive health by using linear regression analyses, crude and mutually adjusted. Included in the analyses were good positive health, Reach, Adoption, and Implementation. Implementation was only included in the analysis for 2020, because it was measured by the change from 2019 to 2020. We cross-checked the analysis using the mean scores of positive health. A *p*-value of <0.05 (two-tailed) was considered statistically significant for all associations. The analyses were performed separately for 2019 and 2020. All quantitative measurements and analyses were carried out with IBM SPSS Statistics 28.

## 3. Results

### 3.1. The implementation of the ISHCs according to the RE-AIM framework

#### 3.1.1. Reach

Reach was measured in the survey according to what extent the ISHCs realized the target of reaching the proportions of 70% female, 70% older, and 70% poor or disadvantaged people. Table 4 gives an overview of ISHCs per province that reached these targets in 2019 and 2020. Most groups in both 2019 and 2020 reached the targets of 70% for participating female, older, and poor or disadvantaged people. Between 60% and 90% in 2019 and 70% and 90% in 2020 of the ISHCs reached the female target. Between 50 and 100% of the ISHCs reached the target of 55 years and older members. The target for poor or disadvantaged people was achieved by 58% in 2019 and 46% of the ISHCs in 2020. In 2020, more ISHCs reached the target set for female and 55 years and older participants compared to 2019.

**Table 4:** Results of ISHCs reaching targets set for female, poor to near poor, and older people participation per provinces for 2019 and 2020.

Year	Reach 70% target	1^a^	2^a^	3^a^	4^a^	5^a^	6^a^	7^a^	8^a^	9^a^	Total
2019 *n* (%)	Female	8 (89%)	6 (60%)	9 (90%)	9 (90%)	10 (71%)	12 (80%)	9 (90%)	6 (60%)	9 (90%)	77 (79%)
	55 years and older	8 (89%)	5 (50%)	10 (100%)	10 (100%)	6 (43%)	11 (73%)	9 (90%)	7 (70%)	7 (70%)	72 (74%)
	Poor or disadvantaged	7 (78%)	2 (20%)	7 (70%)	7 (70%)	5 (36%)	12 (80%)	6 (60%)	6 (60%)	3 (30%)	56 (58%)
2020 *n* (%)	Female	8 (89%)	8 (80%)	7 (70%)	9 (90%)	12 (86%)	13 (87%)	7 (70%)	8 (80%)	9 (90%)	80 (83%)
	55 years and older	7 (78%)	7 (70%)	9 (90%)	10 (100%)	7 (50%)	11 (73%)	8 (80%)	10 (100%)	8 (80%)	76 (78%)
	Poor or disadvantaged	4 (44%)	4 (40%)	2 (20%)	6 (60%)	5 (36%)	9 (60%)	6 (60%)	7 (70%)	3 (30%)	45 (46%)

^a^Provinces: 1. Bac Ninh (*n* = 9)/ 2. Hai Duong (*n* = 10)/ 3. Hai Phong (*n* = 10)/ 4. Hanoi (*n* = 10)/ 5. Hoa Binh (*n* = 14)/ 6. Hung Yen (*n* = 15)/ 7. Ninh Binh (*n* = 10) /8. Thai Binh (*n* = 10)/ 9. Vin Phuc (*n* = 10).

#### 3.1.2. Effectiveness

Effectiveness was measured in the FGDs by satisfaction and further experiences with participation in the clubs. In the FGDs, participants expressed being very satisfied with the ISHCs, with almost 75% of participants rating their satisfaction between 9 and 10. Participants in the urban and semi-urban areas gave the highest scores for satisfaction compared to the other areas. Table 5 gives an overview of the positive and challenging experiences of the participants per area. In the coastal and rural areas, economic development for the members and for the community as whole is mentioned as a positive outcome of their membership. In the mountainous, urban, and semi-urban areas, the participants mentioned that membership improved their social life and feeling of unity and solidarity by helping others in the community. Other positive outcomes mentioned by the participants were feeling healthier, having a more active lifestyle, feeling more confident, and enjoying cultural and social activities due to membership. In the mountainous and one of the semi-urban areas, the high rate of female participants leading to stronger female opinions is mentioned as a challenge. In the rural area, the participants mentioned that certain groups saw limited benefits, such as disabled and disadvantaged people, due to lack of (financial) resources. Other challenges mentioned by the participants were the varying commitment and participation of the members and the loans not being high enough.

**Table 5:** Overview of experiences with ISHCs membership.

Area	Experiences with membership
Coastal	Positive: Health improvement due to healthier lifestyle (more exercise, healthier diet, and check-ups). More sharing and exchange between male and female community members. More opportunities due to loan attainment. Challenging: The continuous participation and commitment of members varied.
Rural	Positive: Social and cultural activities result in happiness. More health knowledge, such as self-care. More economically developed. Challenging: Limited benefits for some groups of people due to the lack of (financial) resources.
Mountainous	Positive: Knowledge about health and rights and entitlement improved. Feeling healthier. Feeling more united by helping the community. Challenging: More female participants compared to male leading to the stronger presence of female opinions.
Semi urban (1)	Positive: Feeling more united by helping others. Feeling more confident and resourceful. The role of older people is emphasized. Challenging: Loans are not high enough.
Semi-urban (2)	Positive: Reaching the disadvantaged people in the community. More economically development in the community. United feelings. Feeling more confident. Challenging: More female participants compared to male leading to the stronger presence of female opinions.
Urban	Positive: Improved social life. Opportunity to help others and social security. More confidence and feeling healthier. Challenging: In a big community, it is impossible to reach everybody.

#### 3.1.3. Adoption

Adoption was measured as the degree to which ISHCs reached targets for 16 indicators (see Supplementary Table S1 for targets). The ISHCs achieved high scores on Adoption, especially for healthcare activities such as physical exercise and healthcare check-ups, namely 74%–91% in 2019 and 79%–98% in 2020 (Supplementary Table S1). The ISHCs in the rural and mountainous areas achieved the highest scores for the adoption of healthcare activities and urban areas the lowest. Moreover, the ISHCs highly achieved targets regarding homecare volunteers supporting the community (2019: 97% and 2020: 99%) and community support (2019: 100% and 2020: 96%). The ISHCs in mountainous areas scored highest on adoption scores for other activities and the ISHCs in urban areas the lowest. The targets set for number of members (55% in 2019 and 60% in 2020) and in 2020 monthly-organized meetings (5%) and sources of income (60%) were reached the least, with the ISHCs in the coastal area scoring relatively better and in the urban area relatively poorly.

#### 3.1.4. Implementation

Implementation was measured by the differences in the reach of the targets for adoption between 2019 and 2020. Implementation of the ISHCs, i.e., ISHCs fidelity including the consistency of adoption scores, improved: more ISHCs reached the targets in 2020 compared to 2019. In total, 61 ISHCs had similar or improving implementation. In particular, homecare volunteers and community support activities increased, with 99% of the ISHCs achieving the targets in 2020. The number of ISHCs reaching targets for healthcare activities decreased slightly in 2020. Furthermore, those organizing monthly meetings dropped from 57% in 2019 to 5% in 2020. The ISHCs in the coastal and urban areas improved the most in 2020 compared to 2019 in achieving their targets and the (semi-)urban areas improved the least.

#### 3.1.5. Maintenance

Organizational maintenance was measured by experienced sustainability and management in the individual interviews and individual maintenance as personal experiences and willingness to retain membership of ISHCs in the FGDs.

##### 3.1.5.1. Organizational

The interviewed participants were confident in the maintenance of the ISHCs (Supplementary Table S2). Most participants indicated that the ISHCs had detailed plans to remain active and to expand to more areas by working on advocacy towards local governments and other associations for funding. All participants indicated the replication of ISHCs in other areas because of spillover effects, e.g., due to members sharing experiences and knowledge with non-members, more areas want to implement the ISHC-model. Expressed concerns were that despite the recognition of their effectiveness by local authorities, resources for funding and training were limited. Mentioned improvements to maintain were organizing activities in the evening for daytime workers and attracting more male participants. One participant indicated the limited loan value in their ISHC, making it unattractive to become a member.

##### 3.1.5.2. Individual

All the participants of the FGDs indicated that the ISHCs could be maintained and sustainable in the future. The members indicated that younger people would follow the older people, in accordance with the Vietnamese saying: “*Young shoots spring up when bamboos grow old.*” The challenges mentioned were the non-ISHCs members not supporting the clubs because they do not understand the purpose, do not like the high female participant rate and the opinions of the female participants, and the lack of resources. To maintain, sustain, and expand the ISHCs, the participants identified the need for more (financial) resources and training of (young) volunteers.

### 3.2. Association of implementation with member-reported positive health

In 2020, the proportion of members reporting good positive health was slightly lower than in 2019 (Supplementary Table S3).

Table 6 presents the associations between the implementation aspects and reported positive health. In 2019, in the crude and mutually adjusted models, the implementation aspect *Adoption* was positively associated with reporting good positive health (crude: B = 2.21; 0.76; 3.66 and mutually adjusted: B = 1.94(0.228;3.643) and B = 1.94(0.234;3.655)). This indicates that the ISHC groups with higher adoption scores (i.e., ISHCs achieving targets on 16 indicators) had more members reporting good positive health. No significant association was found for *Reach* and good positive health. In 2020, no significant associations were found for *Reach*, *Adoption*, or *Implementation* and reported positive health.

**Table 6:** Associations of implementation aspects with reported good positive health: results of regression analyses leading to regression coefficients (B) and 95% confidence intervals.

	Crude^a^ B (95% CI)	Adjusted^b^ B (95% CI)
Implementation aspects 2019^a^		
Reach	−1.95 (−6.915;3.010)	−2.02 (−6.882;2.838)
Adoption	1.94 (0.228;3.643)*	1.94 (0.234;3.655)*
Implementation aspects 2020^b^		
Reach	4.90 (−1.510;11.317)	4.42 (−2.026;10.856)
Adoption	−0.861 (−3.916;2.193)	−1.87 (−5.226;1.517)
Implementation	7.415 (−3.329;18.158)	10.895 (−1.453;22.360)

*Significant by *p* < 0.05.

a Crude analysis: bivariate analysis.

b Mutually adjusted analysis: multivariate analysis.

The results of cross-checking the association of implementation aspects and the mean score of positive health were similar (Supplementary Table S4).

## 4. Discussion

We assessed the implementation of the Older People Associations (OPA) model in Vietnam with the Reach, Effectiveness, Adoption, Implementation, and Maintenance (RE-AIM) framework and the association of the degree of implementation with members reporting good positive health. We found that the *Reach* of the Intergenerational Self-Help Clubs (ISHCs) was adequate for poor, female, and older people. Members were very satisfied with ISHCs, showing high *Effectiveness*. *Adoption* was similarly adequate, especially for healthcare activities, and *Implementation* increased over time. Moreover, *Maintenance* was good and, regarding sustainability, promising. The ISHCs improved on all RE-AIM framework aspects in 2020 compared to 2019. We further found that higher adoption was associated with more frequent reporting of having good positive health in 2019.

This study shows good implementation of the OPA model and multiple benefits for its members. This corresponds with other research on the OPAs showing positive effects on multiple levels of members’ lives (14). Moreover, the finding that training and education is important for the maintenance and sustainability of ISHCs confirms findings on other community-based interventions in other Southeast Asian countries (17, 18). However, to our knowledge, this is the first study to evaluate the implementation of ISHCs with the RE-AIM framework, in which group-level and member-level outcomes of the ISHCs were studied with a mixed-method approach. Using the RE-AIM framework with qualitative and quantitative data on individual- and organizational-level outcomes corresponds with what is done in the ISHCs and has provided evidence for scaling up these community-based approaches. This contributes to the applied research and literature on the RE-AIM framework and community-based approaches in general.

Regarding vulnerable groups, we found that the OPA model reaches people that are most vulnerable to the arising issues of ageing, namely, older, female, disabled, and disadvantaged people. In Vietnam, the care of older people is mostly provided by the family (17), and children are often seen as a guarantee of older age security. However, this ageing trend in Vietnam is jeopardizing this traditional safety net as there are more older persons with chronic diseases in need of support and care. The demand for non-family support and caregiving for older persons is increasing, and the availability of social services to meet this demand is limited (17). In the OPAs, this demand is partly met by delivering homecare to the most vulnerable members and non-members by providing health check-ups and health promotion and education. Moreover, the OPAs promote older people’s participation in communities, the inclusion of older people with disabilities, and the participation and leadership of older female people, as well as for non-members (19). This corresponds with our findings that the ISHCs meet this demand by highly adopting healthcare and community support activities and showing improvement in perceived health.

For 2019, we found that the ISHCs with higher adoption scores had more members reporting good positive health, but we could not confirm this for 2020 even though adoption improved in this year. A possible explanation for this finding could be that the data for this study were collected during the COVID-19 outbreak in 2020 in the northern region of Vietnam, which had the highest rate of cases and deaths due to the pandemic (20, 21). This could have halted the improvement of perceived health. Research has associated the COVID-19 restrictions with psychological impacts such as depression and anxiety (21). This is especially the case for older people as in the early stage of the COVID-19 outbreak, the disease had been predominantly portrayed as affecting mostly older adults, leading to the social marginalization and segregation of older people (22). The ISHCs include relatively many older people, and the halting of the improvement of perceived health could reflect that. Moreover, in 2020, the ISHCs organized fewer monthly sessions, most likely due to COVID restrictions. This could have affected the reporting change in perceived health due to less active participation in the ISHCs.

### 4.1. Strengths and limitations

A strength of this study is that we used multiple quantitative and qualitative data sources, allowing triangulation, which strengthens the validity of the findings. Another strength is the use of data from multiple ISHCs, covering multiple provinces over 2 years, and the possibility of linking the board surveys with the members of the same ISHCs.

A limitation is our use of self-report data, which may have introduced information bias, i.e., giving socially desirable information due to feeling the need to maintain a positive and harmonious relationship with the interviewer (22). However, data were filled in anonymously, reducing the likelihood of such bias. In addition, the study was set up for internal evaluation, not as a longitudinal study, meaning individual changes could not be measured.

### 4.2. Implications

We found that ISHC-model reaches the most vulnerable people to arising issues in Vietnam, such as a high incidence of disability and chronic diseases, poor access to healthcare, higher medical costs, low coverage of social protection, and insufficiency of family support (23), which implies that the ISHC-model is promising to promote health and to reach several vulnerable groups. To further confirm the applicability of the ISHCs, future research in other regions is needed.

Our research shows how the RE-AIM framework can be used for evaluating the implementation of community-based approaches. Community-based programs are real-world settings (24), and therefore, the RE-AIM framework is seldomly applied (25). This addresses a significant gap in implementation science and RE-AIM literature, providing a valuable tool for funders and researchers to have an insight into real-world evaluations (26). We encourage researchers to use a similar approach of researching the implementation of health promotion in community-based programs.

We found high confidence in the maintenance of the clubs; however, to maintain self-help community-based interventions such as the ISHCs, training and training resources are highly important (27). To impart stronger conclusions about the clubs and their training, we suggest studying the effectiveness of the training and the materials. Research into the effectiveness of the training can provide stronger conclusions about the ISHCs and their effectiveness. We found that future funding of the ISHCs is seen as a challenge for maintenance, possibly having implications for sustainability. The ISHCs are financed by a combination of loan interest, membership fees, collective income generation, and external funding by (mostly) non-governmental organizations. These findings can strengthen the awareness of financial and resource support from local agencies.

We were the first to assess the implementation of ISHCs using the RE-AIM framework, including facilitators and barriers, during the COVID-19 pandemic. This implies a need to confirm our findings by other studies. Preferably, such a replication study would make use of longitudinal data and cover other countries in Asia too as the OPA-model has been implemented widely. This can support policies to improve the positive health of older people throughout Asia.

## 5. Conclusion

This study showed that the implementation of community-based programs in Vietnam is promising for promoting public health. The OPA model is a feasible community-based support program that has been successfully implemented in Vietnam. The results show the ISHCs reach a vast majority of female, older, and disadvantaged people and lead to high member satisfaction, high adoption of healthcare and community activities, high confidence in maintenance, and improvement of implementation. This study shows the potential of the OPA model for improving the health of older people and of the RE-AIM framework for assessing the implementation of community health promotion approaches.

## Data availability statement

The original contributions presented in the study are included in the article/Supplementary materials, further inquiries can be directed to the ZP, z.e.pardoel@umcg.nl.

## Ethics statement

The studies involving human participants were reviewed and approved by HelpAge International. The patients/participants provided their written informed consent to participate in this study.

## Author contributions

NV and LP were involved in the preparation and data collection. ZP, JK, and JL conceptualized the study. ZP led the data analysis, wrote the initial draft of the manuscript, produced the tables, and incorporated contributing author feedback into the paper. JL, SR, RL, and MP contributed to all the drafts and manuscript. All authors contributed to the article and approved the submitted version.

## Funding

This work was supported by the European Union’s Horizon 2020 research and innovation program called SC1-BHC-16-2018 Global Alliance for Chronic Diseases (GACD)—Scaling-up of evidence-based health interventions at population level for the prevention and management of hypertension and/or diabetes, soliciting for research in Low- and Middle-Income Countries (LMIC), under grant agreement No: 825026. The funding source was not involved in the data collection and analysis nor the writing and publication of the manuscript.

## Conflict of interest

The authors declare that the research was conducted in the absence of any commercial or financial relationships that could be construed as a potential conflict of interest.

## Publisher’s note

All claims expressed in this article are solely those of the authors and do not necessarily represent those of their affiliated organizations, or those of the publisher, the editors and the reviewers. Any product that may be evaluated in this article, or claim that may be made by its manufacturer, is not guaranteed or endorsed by the publisher.

## Abbreviations

ISHCs: Intergenerational Self-Help Clubs
OPA: Older People Associations
RE-AIM: Reach, Effectiveness, Adoption, Implementation, and Maintenance
FGD: Focus group discussion
e.g.: exempli gratia—Latin phrase meaning “for example”
i.e.: *id est*—Latin phrase meaning “that is”
