# Supplementary material for: The implementation of community-based programs in Vietnam is promising in promoting health
Source: Front Public Health. 2023 Jun 20;11:1182947. doi: 10.3389/fpubh.2023.1182947 (PMC10322193; doi:10.3389/fpubh.2023.1182947)
Supplement: Supplementary file 1 [file Table_1.pdf]

1. Description and results Reach and Adoption

To reach the target groups, the ISHC-model follows the 70-70-70 formula: 70% of all members ought to be 55 years and older, 70% is ought to be female and 70% are expected to either being disadvantaged, near poor, or poor. For Adoption, the results per indicator are presented in table A.

Tabel A Percentage of ISHCs per province reaching targets set for Adoption indicators with legend

Year	Category	Adoption indicator	1*	2*	3*	4*	5*	6*	7*	8*	9*	Total
2019	*General*	*a. Number of members*	78%	30%	90%	50%	15%	67%	60%	60%	50%	55%
		b. Member attendance	67%	70%	60%	20%	100%	100%	50%	80%	80%	72%
		c. Monthly meetings	56%	40%	80%	40%	69%	67%	50%	50%	50%	57%
		d. Sources of income	100%	70%	100%	80%	92%	67%	90%	100%	50%	83%
	Healthcare	*e. Members practicing physical exercise*	89%	100%	70%	90%	100%	100%	100%	90%	100%	97%
		f. Health check-ups per year	100%	90%	80%	70%	100%	100%	90%	70%	90%	88%
		g. Member receiving health-checkup	89%	100%	80%	60%	100%	100%	90%	70%	60%	80%
		h. Health session for members	89%	40%	80%	80%	92%	93%	50%	70%	80%	74%
		i. Health sessions for non-members	100%	90%	90%	40%	77%	93%	100%	80%	90%	87%
		j. Non-members participating in health sessions	100%	90%	90%	50%	92%	100%	100%	90%	90%	91%
	Other activities	k. Income generating activities	56%	50%	70%	30%	100%	87%	70%	80%	50%	68%
		l. Homecare volunteers supporting people	100%	100%	100%	90%	100%	100%	90%	90%	100%	97%
		m. People receiving support from homecare volunteers	67%	100%	70%	70%	100%	100%	70%	100%	90%	87%
		n. Community support activities	56%	40%	80%	90%	77%	67%	40%	70%	80%	67%
		o. Members receive community support	100%	100%	100%	100%	100%	100%	100%	100%	100%	100%
		p. Rights and Entitlement sessions	56%	80%	80%	80%	100%	100%	60%	90%	80%	81%
		**Adoption indicators**	**1[a]**	**2[a]**	**3[a]**	**4[a]**	**5[a]**	**6[a]**	**7[a]**	**8[a]**	**9[a]**	**Total**
2020	*General*	*a. Number of members*	89%	40%	90%	40%	31%	67%	60%	80%	50%	60%
		b. Member attendance	78%	90%	90%	80%	100%	100%	70%	100%	100%	91%
		c. Monthly meetings	0%	0%	0%	0%	0%	0%	0%	50%	0%	5%
		d. Sources of income	44%	50%	70%	70%	46%	73%	60%	80%	40%	60%
	Healthcare	*e. Members practicing physical exercise*	100%	100%	90%	100%	92%	100%	100%	100%	100%	98%
		f. Health check-ups per year	89%	100%	40%	70%	92%	100%	100%	80%	100%	87%
		g. Member receiving health check-up	67%	100%	60%	60%	92%	100%	80%	100%	100%	86%
		h. Health session for members	89%	100%	50%	80%	92%	100%	100%	80%	100%	89%
		i. Health sessions for non-members	100%	40%	60%	40%	92%	100%	100%	70%	100%	79%
		j. Non-members participating in health sessions	100%	90%	80%	80%	92%	100%	100%	70%	100%	91%
	Other activities	k. Income generating activities	56%	90%	20%	10%	92%	73%	100%	80%	100%	70%
		l. Homecare volunteers supporting people	89%	100%	100%	100%	100%	100%	100%	100%	100%	99%
		m. People receiving support from homecare volunteers	67%	90%	100%	100%	100%	100%	100%	100%	100%	96%
		n. Community support activities	78%	100%	100%	100%	54%	100%	100%	100%	100%	92%
		o. Members receive community support	100%	100%	100%	90%	100%	100%	100%	100%	100%	99%
		p. Rights and Entitlement sessions	33%	90%	80%	20%	100%	93%	100%	90%	100%	80%

∗Provinces: 1. Bac Ninh (n=9)/ 2. Hai Duong (n=10)/ 3. Hai Phong (n=10)/ 4. Hanoi (n=10)/ 5. Hoa Binh (n=14)/ 6. Hung Yen(n=15)/ 7. Ninh Binh(n=10) /8. Thai Binh(n=10)/ 9.Vin Phuc(n=10)

Legend for targets set per indicator

General targets:

a. 55 members

b. 85% attendance

c. 12 monthly meetings per year

d. 3 income sources

Healthcare targets:

e. 85% members at least three times a week: 85%

f. 2 Health check-ups per year

g. 90% members at least twice a year

h. 4 health session for members per year

i. 2 health sessions for non-members per year

j. 100 non-members per year

Other activities targets:

k. 4 Income generating activities per year

l. 10 volunteers supporting people twice a week

m.5 persons that receive support from homecare volunteers

n. 1 community support activities per month

o. 2 members receive community support monthly

p. 2 Rights and Entitlement sessions per year
