# Supplementary material for: The implementation of community-based programs in Vietnam is promising in promoting health
Source: Front Public Health. 2023 Jun 20;11:1182947. doi: 10.3389/fpubh.2023.1182947 (PMC10322193; doi:10.3389/fpubh.2023.1182947)
Supplement: Supplementary file 2 [file Table_2.pdf]

2. Findings on maintenance with quotes from the interviews and focus group discussions

Table B The findings on maintenance with quotes from the interviews and focus group discussions

Theme	*Data source*	*Participant*	*Quotes*
Positive	*Interviews*	Chairperson	*"Not only members but also the community enjoy benefits. Older people, who are lonely, disabled, seriously ill, have been visited by volunteers at home, encouraged and helped with the household. From the success of the ISHC model, many localities have expanded the ISHCs with a great spillover effect."*
	Interviews	Health centre official	*"Local agencies consider continuing implementing the policy of replicating the ISHC model in the coming years. After the funding ends, the city management will assign tasks to manage, guide and monitor activities."*
	FGD	*Participant from mountainous area*	*"During the establishment, we did not understand the nature or function of ISHC; but after a few months of specific activities, we have understood and changed our mind. It has been now stable and already put into operation effectively so the outside community is very excited and wants to join ISHC.*
	FGD	*Semi-urban area participant*	*I find the activities of this club very practical and very humane. Because we can do charity work, help each other, have the support of a volunteer team, we should promote more and replicate and maintain the club."*
	FGD	*Coastal area participant*	*"We feel that joining the club is very beneficial, so if the club does not get support anymore, we still maintain to help each other learn, help each other about culture and arts, together develop the movement and have fun."*
	FGD	*Rural area participant*	*At first, it was difficult because we could not mobilize members. We are old, without salary nor money. We were also afraid of not raising enough capital to establish the club. But after we joined the club, attended the meetings, and understood the club, we encouraged each other to participate, contributing capital to provide enough reciprocal capital for the club."*
Challenges	*Interviews*	Chairperson	*"The need to expand the ISHC model is increasing while local resources are limited or some localities have not made the proper investments in the ISHC due to other priorities. Some localities are still unfamiliar with this new model and need time to adapt to it."*
	Interviews	Health official	*"Local authorities also recognize the effectiveness of the model, making the replication of the club model showing signs of prosperity. But the conditions for funding and training techniques are limited."*
	FGD	Coastal area participant	*"At first, the public said bad words, such as how can the older men go singing and dancing for nothing. People were not aware. Therefore, there is nothing to blame them. This is the club. We are confident to have each other. Those who say that are only a minority."*
	FGD	*Mountainous area participant*	*"In the club, the opinions given by the female members are much more than those given by the male members because the rate of female members is higher than that of the male members."*
	FGD	Rural area participant	*"The limitation of the club is the resources to organise economic, cultural and artistic activities, so some groups of people are limited in benefiting."*
Improvements	*Interviews*	*Official Fatherland Front*	*"Looking forward to the evening activities because the members will attend more fully the evening activities"*
	Interviews	Health centre official	*"The loan value is limited; not significant enough for income increase. In the city, they do not know much what to do with this capital amount to increase their income. This made me want to reduce the number of members to increase the amount of capital.*
	FGD	Semi-urban area participant	*"There should be more support: funding, equipment, such as radio speakers and iron board bracket."*
	FGD	Mountainous area participant	*"In the immediate future, we must educate the young volunteers in the understanding of the programs and content of activities for them to maintain."*
