# Supplementary material for: The implementation of community-based programs in Vietnam is promising in promoting health
Source: Front Public Health. 2023 Jun 20;11:1182947. doi: 10.3389/fpubh.2023.1182947 (PMC10322193; doi:10.3389/fpubh.2023.1182947)
Supplement: Supplementary file 3 [file Table_3.pdf]

3. Results of member surveys on the variables included as the dimensions of positive health

Table C Results of member surveys on the variables included as the dimensions of positive health

	2019										2020									
**Variables**[a]	**1**[b]	**2**[b]	**3**[b]	**4**[b]	**5**[b]	**6**[b]	**7**[b]	**8**[b]	**9**[b]	**Total**	**1**[b]	**2**[b]	**3**[b]	**4**[b]	**5**[b]	**6**[b]	**7**[b]	**8**[b]	**9**[b]	**Total**
**Health status** n(%)	541 (98.3)	463 (88.9)	365 (65.4)	394 (66.6)	672 (95)	816 (99.3)	537 (91.5)	181 (47.8)	456 (87)	4425 (84.4)	437 (76.1)	480 (88.9)	442 (77.5)	510 (83.7)	429 (63.4)	616 (71.5)	471 (77.5)	548 (85.2)	279 (52.6)	4212 (75.1)
**Level of confidence** n(%)	502 (91.3)	519 (99.6)	558 (100)	592 (100)	707 (100)	822 (100)	585 (99.7)	376 (99.2)	524 (100)	5231 (99.8)	572 (99.7)	540 (100)	551 (96.7)	606 (99.5)	675 (99.7)	861 (100)	605 (99.5)	641 (99.7)	525 (99.1)	5576 (99.4)
**Feeling of unity/ solidarity** n(%)	550 (100)	521 (100)	558 (100)	590 (99.7)	707 (100)	822 (100)	587 (100)	375 (98.9)	524 (100)	5236 (99.9)	574 (100)	537 (99.4)	562 (98.6)	605 (99.3)	675 (99.7)	861 (100)	605 (99.5)	637 (99.1)	530 (100)	5586 (99.5)
**Quality of Life** n(%)	550 (100)	521 (100)	558 (100)	591 (100)	707 (100)	822 (100)	585 (99.7)	377 (99.5)	524 (100)	5235 (99.9)	574 (100)	537 (100)	555 (97.4)	609 (100)	676 (99.9)	852 (99.0)	607 (99.8)	640 (99.5)	530 (100)	5580 (99.4)
**Disability status**[c] n(%)	1: 81 (69.3)	1:68 (51.4)	1: 239 (42.8)	1:258 (43.6)	1:419 (59.3)	1:452 (55.0)	1:352 (60.0)	1:66 (17.4)	1:282 (53.8)	1:2717 (51.9)	1:487 (84.8)	1:419 (77.6)	1:380 (66.7)	1:389 (63.9)	1:477 (70.5)	1:699 (81.2)	1:486 (79.9)	1:490 (76.2)	1:387 (73.0)	1:4214 (75.1)
	2:158 (28.7)	2:212 (40.7)	2:225 (40.3)	2:285 (48.1)	2:269 (38.0)	2:278 (33.8)	2:167 (28.4)	2:267 (70.4)	2:233 (44.5)	2:2094 (40.0)	2:83 (14.5)	2:119 (22.0)	2:160 (28.1)	2:205 (33.7)	2:177 (26.1)	2:155 (18.0)	2:115 (18.9)	2:133 (20.7)	2:141 (26.6)	2:1288 (23.0)
	3:11 (2.0)	3:41 (7.9)	3:88 (15.8)	3: 49 (8.3)	3:19 (19)	3:90 (10.9)	3:66 (11.2)	3:45 (11.9)	3:9 (1.7)	3:418 (8.0)	3:4 (0.7)	3:2 (0.4)	3:24 (4.2)	3:15 (2.5)	3:18 (2.7)	3:7 (0.8)	3:7 (1.2)	3:15 (2.3)	3:2 (0.4)	3:94 (1.7)
			4: 6 (1.1)			4: 2 (0.2)	4: 2 (0.3)	4: 1 (0.3)		4:11 (0.2)			4:6 (1.1)		4:5 (0.7)			4:5 (0.8)		4:16 (0.3)
**Rights and entitlement** n(%)	550 (100)	520 (99.8)	58 (98.2)	591 (99.8)	707 (100)	822 (100)	576 (98.1)	373 (98.4)	524 (100)	5211 (99.4)	568 (99.0)	534 (98.9)	548 (96.1)	609 (100)	676 (99.9)	858 (99.7)	605 (99.5)	635 (98.9)	524 (98.9)	5557 (99)
**Satisfaction**[d] n(%)	4:29 (5.3)	4: 11 (2.1)	4: 80 (14.3)	4: 63 (10.6)	4: 13 (1.8)	5:822 (100)	3: 3 (0.5)	3:3 (0.8)	4: 40 (7.6)	3:6 (0.1)	4:34 (5.9)	3:2 (0.4)	3:6 (1.1)	3:4 (0.7)	3:2 (0.3)	4:98 (11.4)	4:24 (3.9)	2:1 (0.2)	4:112 (21.1)	2:1 (0.1)
	5:521 (94.7)	5:510 (97.9)	5:478 (85.7)	5:529 (89.4)	5:694 (98.2)		4: 44 (7.5)	4: 98 (25.9)	5:484 (92.4)	4: 378 (7.2)	5:540 (94.1)	4:21 (3.9)	4:65 (11.4)	4:47 (7.7)	4:58 (8.6)	5:763 (88.6)	5:584 (96.1)	3:2 (0.3)	5:418 (78.9)	3:16 (0.3)
							5:540 (92.0)	5:278 (73.4)		5:4856 (92.7)		5:517 (95.7)	5:499 (87.5)	5:558 (91.6)	5:617 (91.1)			4:35 (5.4)		4:494 (8.8)
																		5:605 (94.1)		5:5101 (90.9)

a. All variables were measured by asking if improved due to participation in the ISHCs

b 1. Bac Ninh/ 2. Hai Duong/ 3. Hai Phong/ 4. Hanoi/ 5. Hoa Binh/ 6. Hung Yen/ 7. Ninh Binh /8. Thai Binh/ 9.Vin Phuc

c Categories: Are you disabled: 1: No, 2: A little, 3: A lot, 4: Totally

d Categories: 1: Not at all, 2: No, 3: A little 4: Yes, 5: Very much
