# Supplementary material for: The implementation of community-based programs in Vietnam is promising in promoting health
Source: Front Public Health. 2023 Jun 20;11:1182947. doi: 10.3389/fpubh.2023.1182947 (PMC10322193; doi:10.3389/fpubh.2023.1182947)
Supplement: Supplementary file 4 [file Table_4.pdf]

4. Results of cross-check regression analyses of implementation aspects and mean Positive Health

Table D presents the associations between implementation aspects and the mean score on positive health. The findings are similar to the regression analyses with number of members reporting positive health, in which in 2019 Adoption is positively associated with the mean score on positive health(crude: B=0.02; 0.000;0.043 and mutually adjusted: B=0.02; 0.000;0.043).

Table D Associations of implementation aspects with reported good Positive Health: results of regression analyses leading to regression coefficients (B) and 95%-confidence intervals

**Implementation aspects 2019**	**Crude[a] B(95% CI)**	**Adjusted[b] B(95% CI)**
Reach	-0,02(-0.085;0.037)	-0.03(-0.085;0.035)
Adoption	0.02(0.000;0.043)*	0.02(0.000;0.043)*
**Implementation aspects 2020**		
Reach	0.04(-0.029;0.117)	0.04(-0.034;0.113)
Adoption	-0.01(-0.042;0.028)	-0.02(-0.057;0.021)
Implementation	0.08(-0.040;0.204)	0.11(-0.025;0.248)

a Crude analysis: bivariate analysis

b mutually adjusted analysis: multivariate analysis

* Significant by $p<0.05$
